# Evaluation of the Efficacy of Semaglutide Dose Escalation in Reducing HbA1c Levels and Insulin Dose in Type 2 Diabetes Patients: Real-World Semaglutide Data from Türkiye, SEMA-TR Study

**DOI:** 10.3390/jcm15114105

**Published:** 2026-05-26

**Authors:** Hilmi Erdem Sumbul, Bektas Isik, Ahmet Gazi Mustan, Irfan Alisan, Cigdem Erhan, Fatma Inci Koca, Aysenur Ucar, Mehmet Erdevir, Begum Seyda Avci, Merve Saracoglu Sumbul, Dilan Damla Ozturk, Mustafa Lutfullah Ardic, Huseyin Ali Ozturk, Fatih Necip Arıcı, Cahit Dincer, Kubilay Akbal, Okan Pirinci, Fadime Koca, Mevlut Koc

**Affiliations:** 1 Department of Internal Medicine, University of Health Sciences, Adana Health Practice and Research Center, Adana 01230, Türkiye; beko44485@gmail.com (B.I.); agazi_mstn@hotmail.com (A.G.M.); dr.irfanalisan@gmail.com (I.A.); begumtngnr@hotmail.com (B.S.A.); drozturkhuseyinali@gmail.com (H.A.O.); fatihneciparici803@gmail.com (F.N.A.); cahitdincer1164@hotmail.com (C.D.); 2 Department of Internal Medicine, Seyhan State Hospital, Adana 01150, Türkiye; drcerhan@gmail.com (C.E.); fatmaincikoca@hotmail.com (F.I.K.); 3 Department of Internal Medicine, Yuregir State Hospital, Adana 01240, Türkiye; dr.aysenurucar@hotmail.com (A.U.); erdevirmehmet@gmail.com (M.E.); dilandamlabal@gmail.com (D.D.O.); 4 Department of Public Health, Adana City Research and Training Hospital, Adana 01230, Türkiye; mervessumbul@gmail.com; 5 Department of Cardiology, University of Health Sciences, Adana Health Practice and Research Center, Adana 01230, Türkiye; drmustafaardic@gmail.com (M.L.A.); mevlutkoc78@yahoo.com (M.K.); 6 Department of Internal Medicine, 5 Ocak State Hospital, Adana 01210, Türkiye; dr.kubilayakbal@hotmail.com; 7 Department of Internal Medicine, Duzici State Hospital, Osmaniye 80600, Türkiye; pirinciokan@gmail.com; 8 Department of Cardiology, Cukurova State Hospital, Adana 01170, Türkiye

**Keywords:** semaglutide, type 2 diabetes mellitus, HbA1c, insulin dose reduction, dose escalation, GLP-1 receptor agonist

## Abstract

**Background**: Several studies have demonstrated that adding semaglutide to the treatment of patients with type 2 diabetes mellitus (T2DM) reduces insulin requirements and glycated hemoglobin (HbA1c) levels. This study aimed to investigate real-world evidence for the effects of semaglutide dose escalation on HbA1c, body weight, dyslipidemia, and insulin dose reduction in patients with T2DM in the Cukurova region of Türkiye. **Methods**: This retrospective cohort study enrolled 500 patients (255 male, 245 female; mean age 56.1 ± 10.8 years) who initiated semaglutide therapy for T2DM between 2024 and 2025. Patients were grouped according to their maximum semaglutide dose: 0.25 mg (Group I), 0.50 mg (Group II), and 1.00 mg (Group III). The primary endpoint was the change in HbA1c from baseline to end of study (30 weeks) across semaglutide dose escalation groups. Secondary endpoints included changes in body weight, frequency of insulin dose reduction, and effects on lipid parameters. **Results**: A total of 117 patients (23.4%) discontinued semaglutide therapy, while 383 patients (76.6%) completed the study. The primary endpoint revealed a mean HbA1c reduction of −1.03 ± 0.35% from baseline to end of study (95% CI 0.99–1.07; t = 58.644; *p* < 0.001). Reductions in HbA1c increased progressively from Group I to Group III (HbA1c: −0.72 ± 0.28, −1.02 ± 0.26, −1.27 ± 0.34%). Insulin dose reduction frequency increased significantly from Group I to Group III (40%, 41%, and 51%, respectively; *p* = 0.010), with a statistically significant difference only between Group I and Group III. At the end of follow-up, rates of hypoglycemic episodes and gastrointestinal (GI) adverse events were similar across groups. **Conclusions**: In a real-world population from the Cukurova region of Türkiye, semaglutide dose escalation in T2DM patients achieved clinically meaningful glycemic control, body weight reduction, LDL-cholesterol lowering, and a significant increase in insulin dose reduction frequency, without a significant increase in GI adverse events.

## 1. Introduction

Uncontrolled glycemic levels represent the most common reason for hospitalization in patients with type 2 diabetes mellitus (T2DM) [1]. For this reason, the American Diabetes Association (ADA) guidelines establish glycemic targets for T2DM patients and recommend that these targets be achieved [1,2]. The typical target glycated hemoglobin (HbA1c) level in T2DM is <7%, although a target of <9% may be considered depending on comorbidities and other patient-specific clinical factors [1,2]. In T2DM management, non-pharmacological interventions (e.g., lifestyle modification) and metformin are the first-line approach; if the HbA1c target is not achieved, insulin, a glucagon-like peptide-1 receptor agonist (GLP-1RA), or a sodium–glucose cotransporter-2 (SGLT2) inhibitor is recommended as a second-line agent [1,2]. Insulin therapy is indicated for patients with blood glucose ≥ 300 mg/dL or HbA1c ≥ 10% at diagnosis. In general, the most pronounced reductions in HbA1c levels have been reported with treatment regimens involving insulin, certain GLP-1 receptor agonists (particularly semaglutide), and tirzepatide, while DPP-4 inhibitors tend to produce comparatively smaller reductions in HbA1c levels [1,2]. The choice of pharmacological therapy is influenced by the patient’s comorbidities, such as coronary artery disease (CAD), heart failure, and chronic kidney disease (CKD).

Clinical evidence demonstrates that GLP-1RAs achieve glycemic control in T2DM patients comparable to insulin, with a lower risk of hypoglycemia [3]. Additional benefits of GLP-1RAs include weight loss, blood pressure reduction, and improvement in lipid profiles. The most common adverse events (AEs) associated with GLP-1RAs are gastrointestinal (GI) in nature. The 2025 ADA guidelines state that the initiation of GLP-1RAs is appropriate in patients already receiving insulin [2]. Although GLP-1RAs alone do not increase hypoglycemia risk, concomitant use of a GLP-1RA with insulin can still lead to hypoglycemia [3]. In such patients, the primary driver of hypoglycemia risk is insulin [4]. Therefore, reducing or discontinuing insulin doses when initiating a GLP-1RA may mitigate this risk.

Beta-cell dysfunction plays a central role in the pathophysiology of T2DM [5]. Progressive decline in beta-cell mass and function is a hallmark of T2DM onset and progression. Semaglutide is a human GLP-1 analogue administered once weekly for the treatment of T2DM. Semaglutide exhibits 94% amino acid sequence homology with endogenous GLP-1 and is structurally related to liraglutide [6,7]. Minor structural modifications reduce its susceptibility to degradation by dipeptidyl peptidase-4 while improving albumin binding [K15]. As a result, semaglutide has a prolonged half-life of approximately one week [6], enabling once-weekly subcutaneous administration [6,7]. Once-weekly semaglutide administered for 12 weeks has been shown to improve beta-cell function and glycemic control in patients with T2DM [8,9]. Accordingly, the addition of semaglutide to existing insulin or oral anti-diabetic (OAD) regimens has been shown to reduce insulin dose requirements and OAD use [9,10,11,12].

Previous real-world studies have reported the beneficial effects of semaglutide treatment on HbA1c levels, body weight, dyslipidemia, and reductions in insulin dose and injection frequency among patients with T2DM [12,13,14,15,16,17,18,19,20,21,22,23]. Nevertheless, based on our literature search, real-world data from Türkiye regarding these outcomes remain limited or unavailable. Given the widespread use of semaglutide in our country, we believed that presenting national real-world data would contribute meaningfully to the literature. Furthermore, therapeutic efficacy and treatment responses may differ across ethnicities and populations, highlighting the importance of population-specific clinical data.

The aim of the present study was to investigate real-world evidence for the effects of semaglutide dose escalation on HbA1c, body weight, dyslipidemia, and insulin dose and frequency reduction in patients with T2DM in the Cukurova region of Türkiye.

## 2. Methods

### 2.1. Study Population

This retrospective cohort study screened patients followed and treated for T2DM at the Department of Internal Medicine outpatient clinic of our institution between 2024 and 2025. T2DM diagnosis and treatment were in accordance with the 2025 ADA Standards of Care [13]. The treatment goal for all included T2DM patients was symptom elimination, prevention and delay of complications, and achievement of glycemic targets. All included patients were receiving at least one OAD, including metformin. Sample size was estimated based on prior studies, with a power analysis performed at 80% power and *p* < 0.05; approximately 400 patients were deemed sufficient. Exclusion criteria were: age < 18 years; type 1 diabetes mellitus; no semaglutide therapy; concurrent use of a dipeptidyl peptidase-4 (DPP-4) inhibitor with semaglutide; severe valvular heart disease; heart failure with reduced ejection fraction; alcohol dependence; inflammatory or hematological disorders; malignancy; active thyroid disease; advanced liver disease; active urinary tract infection; advanced-stage CKD; and pregnancy or suspected pregnancy.

The study protocol was approved by the Clinical Research Ethics Committee of the University of Health Sciences Adana Health Practice and Research Center (Ethics Committee No: 2026/01/15-1051). The Ethics Committee waived the requirement for informed consent for retrospective clinical data analysis; therefore, informed consent was not obtained from the study participants. Following the application of the exclusion criteria to all patients screened for eligibility, 167 patients were excluded. The final study population consisted of 500 patients with type 2 diabetes mellitus who met all inclusion criteria, had initiated semaglutide treatment, were regularly followed during the study period, and had complete data available for all evaluated parameters.

### 2.2. Demographic, Clinical, and Laboratory Assessment

All T2DM patients screened for the study underwent a detailed file review and clinical assessment for inclusion and exclusion criteria. Patients’ demographic, clinical, and laboratory data were retrospectively obtained from the electronic medical record systems routinely used by healthcare professionals in our hospital and from the nationally integrated e-Nabız (https://enabiz.gov.tr) healthcare database. In addition, the initiation timing of semaglutide therapy and subsequent dose adjustments were monitored through these systems. Demographic information, medical history, and physical examination findings were recorded. Age, sex, smoking status, hypertension (HT), CAD, CKD, and dyslipidemia were documented. Baseline weight, waist circumference, and body mass index (BMI) prior to semaglutide initiation were recorded. Venous blood samples were collected from the antecubital vein after a 20-min rest period in the supine position. Blood specimens were obtained in tubes containing ethylenediaminetetraacetic acid (EDTA) and centrifuged at 3000 rpm for 10 min at 0 °C. Fasting plasma glucose, HbA1c, total cholesterol, HDL cholesterol, LDL cholesterol, and triglyceride levels were analyzed using standard automated laboratory methods with an automated chemistry analyzer (Abbott Aeroset, Abbott Cardiovascular, 5050 Nathan Lane North, Plymouth, MN 55442, USA) and commercially available assay kits (Abbott). Serum NT-proBNP concentrations were measured using the same analyzer and corresponding commercial kits. Albuminuria assessment was performed using morning urine samples, and urinary albumin-to-creatinine ratio (uACR) values were calculated from laboratory measurements. At 30 weeks of follow-up, weight, waist circumference, BMI, fasting blood glucose, HbA1c, total cholesterol, HDL cholesterol, LDL cholesterol, TG, NT-proBNP, and uACR were re-assessed and recorded.

### 2.3. Semaglutide Treatment

Semaglutide injections were administered subcutaneously into the thigh, abdomen, or upper arm, with rotation of injection sites recommended to minimize local adverse effects. Treatment was administered once weekly on the same day of the week throughout the study period, irrespective of meals. Patients initiated therapy with 0.25 mg once weekly and subsequently underwent dose escalation at four-week intervals until either the target dose of 1.0 mg/week or the maximum tolerated dose was achieved. The escalation strategy was determined according to safety profile, efficacy outcomes (mainly weight reduction), and gastrointestinal tolerability, based on evidence and exposure–response modeling from previous clinical studies in overweight and obese populations.

All patients were encouraged to adhere to the dose-escalation protocol and achieve the target dose whenever possible. In patients with T2DM receiving semaglutide therapy, an HbA1c target below 7% was considered an acceptable glycemic goal.

### 2.4. Follow-Up

Enrolled T2DM patients were followed at 3–6 month intervals over 30 weeks. At each visit, biochemical and clinical parameters were evaluated in accordance with current guidelines. Patients were monitored for: (i) treatment discontinuation (due to GI side effects, non-compliance, increased cost, or access problems); (ii) GI adverse events (nausea, vomiting, diarrhea, and constipation); and (iii) insulin dose reduction, insulin injection frequency reduction, and OAD drug reduction. Follow-up continued for 30 weeks. The primary endpoint was the change in HbA1c from baseline to end of study (30 weeks) across semaglutide dose escalation groups. Secondary endpoints included changes in body weight, frequency of insulin dose reduction, and effects on lipid parameters.

### 2.5. Statistical Analysis

Continuous variables are expressed as mean ± standard deviation (SD), and categorical variables are expressed as frequencies and percentages. Interobserver and intraobserver variability for measurement parameters was assessed using the kappa coefficient. Distribution of continuous variables was evaluated using the Kolmogorov–Smirnov test. For comparisons among three groups, one-way ANOVA was used for normally distributed variables, and the Kruskal–Wallis one-way ANOVA was applied when normality was not confirmed. To compare changes in parameters from baseline to 30-week follow-up in T2DM patients receiving semaglutide therapy, the paired *t*-test was used for normally distributed variables, and the Wilcoxon signed-rank test was used for non-normally distributed variables. Multivariate logistic regression analysis was performed to identify independent predictors of HbA1c < 7% and insulin dose reduction groups. A *p* value of <0.05 was considered statistically significant. All analyses were performed using SPSS version 23.0 (SPSS for Windows, Chicago, IL, USA).

## 3. Results

Of 658 T2DM patients screened, 500 who met all criteria were enrolled (255 male, 245 female; mean age 56.1 ± 10.8 years). Cohen’s kappa values for interobserver variability exceeded 90% for all body measurements (*p* < 0.001). Patients were categorized according to the maximum semaglutide dose achieved in order to evaluate the impact of semaglutide dose escalation on glycemic control and treatment-related outcomes in patients with T2DM: Group I (0.25 mg, *n* = 97), Group II (0.50 mg, *n* = 255), and Group III (1.00 mg, *n* = 148). All patients were followed for 30 weeks for semaglutide-related adverse events, treatment discontinuation and its causes, and changes in T2DM pharmacotherapy. In patients who completed follow-up, baseline and end-of-study clinical and laboratory data were compared. Insulin dose reduction, reduction in insulin injection frequency, and OAD drug reduction were also evaluated.

### 3.1. Baseline Clinical, Demographic, and Laboratory Variables According to Semaglutide Dose

Baseline clinical, demographic, and laboratory data stratified by semaglutide dose group are presented in Table 1. Clinical, demographic, and laboratory variables were similar across all semaglutide dose groups, with the exception of fasting blood glucose. Fasting glucose increased progressively from Group I to Group III; patients in Group I had significantly lower fasting glucose than those in Group III. Glucose levels were similar between the other group pairs. Of the 500 enrolled patients, 202 (40.4%) were receiving insulin therapy at baseline. During follow-up, 117 patients (23.4%) discontinued treatment: 42 (8.4%) due to GI side effects, 9 (1.8%) due to non-compliance, and 66 (13.2%) due to cost or access-related reasons (Table 1).

### 3.2. Follow-Up Clinical, Medical Treatment, and Laboratory Variables According to Semaglutide Dose in Patients Continuing Semaglutide Treatment

After excluding the 117 patients who discontinued treatment, follow-up clinical, medical treatment, and laboratory data for the remaining 383 patients, stratified by semaglutide dose group, are presented in Table 2. Significant differences among groups were observed in weight, absolute weight change, BMI, absolute BMI change, HbA1c, absolute HbA1c change, LDL cholesterol, and absolute LDL cholesterol change, as well as in the frequency of weight loss ≥ 5%, weight loss ≥ 10%, HbA1c < 7%, insulin dose reduction, and insulin injection reduction.

Follow-up weight, BMI, HbA1c, and LDL cholesterol decreased progressively from Group I to Group III; Group III patients had significantly lower values than those in Groups I and II, while Groups I and II did not differ significantly. Absolute weight change, absolute BMI change, absolute HbA1c change, absolute LDL cholesterol change, weight loss ≥ 5%, and weight loss ≥ 10% all increased progressively from Group I to Group III, with significant differences across all groups. The frequency of HbA1c < 7% also increased from Group I to Group III, but reached statistical significance only between Groups I and III.

### 3.3. Insulin Dose Reduction—A Key Clinical Benefit of Dose Escalation

One of the most clinically important findings of this study is the dose-dependent effect of semaglutide on insulin requirements. Insulin dose reduction and insulin injection reduction frequency both increased progressively from Group I to Group III. Specifically, insulin dose reduction was achieved in 40% of Group I patients, 41% of Group II patients, and 51% of Group III patients—a statistically significant trend (*p* = 0.010). Insulin injection frequency reduction followed a similar pattern (32%, 39%, and 46%, respectively; *p* = 0.045), with a significant difference between Group I and Group III. Importantly, this insulin dose reduction was achieved without a corresponding increase in hypoglycemic episodes across groups (*p* = 0.876), underscoring the safety of the approach. These findings strongly support the clinical value of escalating semaglutide to the maximum tolerated dose in insulin-treated T2DM patients, as higher doses not only improve glycemic control but also facilitate meaningful reductions in insulin burden.

The frequency of OAD drug reduction increased progressively from Group I to Group III but did not reach statistical significance across groups. At the end of 30-week follow-up, GI adverse events were observed in 141 patients (36.8%). Although GI adverse event frequency increased from Group I to Group III, the difference was not statistically significant across groups. Only 23 patients (6%) experienced hypoglycemic episodes, with no significant difference among groups. Other clinical and laboratory variables were similar across all groups (Table 2). Among patients with T2DM who continued semaglutide treatment, regression analyses were conducted to evaluate whether semaglutide dose groups independently predicted insulin dose reduction and achievement of HbA1c levels below 7%. The results indicated that semaglutide dose was not an independent determinant of either outcome (*p* = 0.453 and *p* = 0.233, respectively).

### 3.4. Comparison Between Baseline and Follow-Up Clinical and Laboratory Variables in Patients Continuing Semaglutide Treatment

Baseline and end-of-study clinical and laboratory parameters were compared in the 383 patients who completed the 30-week follow-up without discontinuing semaglutide (Table 3). At follow-up, significant reductions from baseline were observed in weight, waist circumference, BMI, fasting glucose, total cholesterol, LDL cholesterol, triglycerides, NT-proBNP, and uACR (all *p* < 0.001). Conversely, HDL cholesterol increased significantly from baseline to end of study (*p* < 0.001).

## 4. Discussion

The present study yielded several important real-world findings, which can be summarized as follows: (i) Semaglutide therapy was associated with significant reductions from baseline in HbA1c, lipid parameters, BMI, NT-proBNP, and uACR, in addition to body weight loss, at 30 weeks. These findings are consistent with previously published data. (ii) HbA1c reduction was evident beginning at the 0.25 mg dose, and progressive semaglutide dose escalation was associated with significantly greater HbA1c reductions without a corresponding increase in GI adverse events, corroborating prior real-world evidence. (iii) Similarly, insulin dose reduction and injection frequency reduction were observed in 41.7% of T2DM patients continuing semaglutide, and both outcomes were further enhanced with dose escalation. (iv) A similar trend was observed for OAD use, with semaglutide dose escalation associated with OAD reduction. (v) Finally, LDL cholesterol reduction was significant from the 0.25 mg dose and continued to increase meaningfully with dose escalation.

### 4.1. Efficacy of Semaglutide on HbA1c

The mean HbA1c reduction of −1.03 ± 0.35% achieved at 30 weeks in our study is consistent with findings from the SURE studies (Germany, Canada, Denmark/Sweden, Switzerland, and the UK) [12,14,15]. The Phase 3 SUSTAIN trials reported mean HbA1c reductions of 1.1% and 1.8% with semaglutide 0.5 mg and 1.0 mg, respectively [16,17,18,19,20,21,22,23,24]. In our study, HbA1c reductions were −0.72 ± 0.28%, −1.02 ± 0.26%, and −1.27 ± 0.34% for the 0.25 mg, 0.50 mg, and 1.00 mg doses, respectively, increasing significantly with escalating doses. The pooled SURE studies reported that 52.6% of 1212 patients reached the target HbA1c < 7% after 30 weeks [15], whereas in our study this proportion was 44.1%. The lower rate of glycemic target attainment in our cohort may be partly attributable to the smaller proportion of patients receiving the 1.0 mg dose (29%). This is supported by the dose-stratified HbA1c < 7% attainment rates of 36.8%, 43.9%, and 49.5% for the 0.25 mg, 0.50 mg, and 1.00 mg groups, respectively. Consistent with previous studies, our findings indicate that achieving the maximum tolerated semaglutide dose may play a critical role in attaining target HbA1c levels during treatment.

### 4.2. Efficacy of Semaglutide on Body Weight

The mean body weight reduction of −6.65 ± 3.22 kg at 30 weeks in our study exceeded that reported in the SURE studies (Germany, Canada, Denmark/Sweden, Switzerland, and the UK) [12,14,15]. The SURE Germany study reported a mean weight reduction of 4.5 kg, while Phase 3 SUSTAIN trials showed reductions of 3.5 kg and 6.5 kg with semaglutide 0.50 mg and 1.00 mg, respectively [12,16,17,18,19,20,21,22,23,24]. In the present study, weight reductions of −4.21 ± 2.68, −6.52 ± 2.84, and −8.59 ± 2.98 kg were observed for the 0.25 mg, 0.50 mg, and 1.00 mg doses, respectively—increasing significantly across escalating doses. The greater weight loss observed in our cohort compared with prior studies may be attributable to the higher baseline body weight of our patients.

### 4.3. Efficacy of Semaglutide on Insulin Dose and OAD Reduction

This section addresses one of the most clinically significant findings of our study. Disease progression in T2DM often necessitates increasing insulin doses, which can promote weight gain. A recent retrospective study of 72 patients receiving high-dose insulin (≥100 units/day) demonstrated that the addition of semaglutide resulted in meaningful reductions in total daily insulin dose alongside HbA1c improvement and weight loss at 6 months [9].

A real-world study with a similar 30-week follow-up period—the SURE Germany study by Menzen et al. [12]—was conducted in 779 T2DM patients, 85.9% of whom completed follow-up. That study reported significant reductions in baseline HbA1c and body weight, but did not specifically evaluate the effect of semaglutide dose escalation on glycemic control or body weight [12]. In the SURE Germany cohort (46% insulin users at baseline), mean total daily insulin dose declined modestly from 57.9 ± 45.78 IU to 56.1 ± 44.31 IU. In our study, insulin dose reduction frequency was significantly higher and demonstrated a clear dose-dependent pattern: 40% in the 0.25 mg group, 41% in the 0.50 mg group, and 51% in the 1.00 mg group (*p* = 0.010). Insulin injection frequency reduction followed the same pattern (32%, 39%, and 46%, respectively; *p* = 0.045). Crucially, this dose-dependent benefit in insulin reduction was achieved without any increase in hypoglycemic episodes across groups (*p* = 0.876), confirming the safety of this approach.

In the broader dose escalation literature, most studies have primarily evaluated glycemic outcomes [10,11,12]. Some have additionally documented insulin dose reductions with escalating doses [10,12], while others have not assessed this outcome [11]. Similarly, data on OAD drug reduction following semaglutide dose escalation are limited [12]. In our study, OAD reduction was observed in 29% of patients receiving escalating semaglutide doses, adding to the sparse evidence on this topic.

Taken together, these findings underscore the clinical importance of escalating semaglutide to the maximum tolerated dose in insulin-treated T2DM patients. Higher semaglutide doses not only yield superior glycemic control and weight loss but also enable meaningful insulin dose reductions—a critical clinical benefit that reduces both the burden of insulin therapy and associated risks such as hypoglycemia and weight gain.

### 4.4. Efficacy of Semaglutide on Lipid Parameters

Prior studies have shown that semaglutide therapy improves fasting and postprandial glucose as well as lipid metabolism [25]. Dyslipidemia is common in T2DM and represents an important risk factor for CAD [26]. One study of 30 patients treated with semaglutide for 12 weeks reported reductions in total and HDL cholesterol but no significant change in LDL cholesterol [25]. A separate study in 65 patients demonstrated significant reductions in total cholesterol, LDL cholesterol, and triglycerides, and a significant increase in HDL cholesterol, at 6 months [26]. Our findings are consistent with the latter study: after 30 weeks of follow-up in 383 patients, significant reductions in total cholesterol, LDL cholesterol, and triglycerides were observed, along with a significant increase in HDL cholesterol. Notably, prior studies have not specifically examined the effect of semaglutide dose escalation on lipid parameters. In our study, dose escalation was significantly associated with LDL cholesterol reduction only; other lipid parameters trended in the expected direction but did not reach statistical significance across dose groups. The SURE Germany study reported significant reductions in total cholesterol, LDL cholesterol, and triglycerides at end of study but no significant increase in HDL cholesterol [12]. The real-world data on the effect of semaglutide dose escalation on lipid parameters provided by our study adds clinically meaningful information to the existing literature.

### 4.5. Efficacy of Semaglutide on NT-proBNP and uACR

NT-proBNP is a biomarker of myocardial stress and a well-established predictor of outcomes in heart failure. Furthermore, when incorporated into multivariable models, NT-proBNP has been shown to incrementally improve the prediction of mortality and cardiovascular events in individuals with type 2 diabetes mellitus (T2DM), particularly in the presence of heart failure (HF), chronic kidney disease (CKD), and recent acute coronary syndrome [27,28,29,30]. Despite this evidence, the use of natriuretic peptides has not yet become fully established in the routine cardiovascular risk assessment of patients with T2DM in clinical practice. In a study by Malachias et al., NT-proBNP alone demonstrated a discriminatory ability comparable to that of multivariable models in predicting both mortality and cardiovascular events among high-risk patients with T2DM and was therefore suggested as a valuable marker for risk stratification [27]. Urinary albumin-to-creatinine ratio (uACR) is an important marker used to predict kidney disease progression and reflect renal function. Patients with uACR levels > 30 mg/g are known to have significantly increased risks of renal progression and cardiovascular events. Elevated uACR is associated with several pathophysiological mechanisms, including endothelial dysfunction, widespread vascular injury, systemic inflammation, altered glomerular hemodynamics, and abnormal tubular function [1,2]. The American Diabetes Association (ADA) Standards of Care recommend maintaining uACR levels below 30 mg/g [1,2]. Previous studies have demonstrated that semaglutide treatment may reduce both NT-proBNP and uACR levels [31,32]. Consistent with the literature, our study also demonstrated reductions in NT-proBNP and uACR levels among patients who continued semaglutide treatment. However, since our study was not designed as a prognostic study, the prognostic significance of these reductions was not evaluated. Additionally, we found that the reductions in NT-proBNP and uACR did not differ significantly among the semaglutide dose groups.

### 4.6. Safety and Adverse Events

An important safety attribute of semaglutide therapy is the absence of an increase in hypoglycemic episodes. In our study, hypoglycemic episode frequency was not affected by semaglutide dose escalation. Prior large-scale real-world data similarly identified GI adverse events as the most common reason for treatment discontinuation, at rates comparable to our findings [12]. The SUSTAIN 5 trial reported treatment discontinuation rates of 6.1% and 9.2% for semaglutide 0.5 mg and 1.0 mg, respectively [20]. In our cohort, GI-related discontinuation was 8.4% overall (4.1%, 8.6%, and 10.8% for 0.25, 0.5, and 1.0 mg, respectively), consistent with these rates. Non-serious adverse events reported in prior studies were 64% and 69% for 0.5 mg and 1.0 mg, respectively [20], compared with 34% and 44% at the same doses in our cohort. Furthermore, dose escalation was not significantly associated with either discontinuation-causing or non-discontinuation adverse events.

### 4.7. Limitations

The most important limitation of this study is its single-center, retrospective design conducted in a single geographic region. Accordingly, the findings of the present study should be interpreted with caution and may not be fully generalizable. Specifically, the study does not provide detailed information regarding adverse events occurring during semaglutide dose escalation, nor does it evaluate the potential effects of dose intolerance, inability to achieve higher doses, or treatment discontinuation on clinical and laboratory outcomes. A prospective, multicenter, randomized study including patients from diverse ethnic backgrounds would provide more generalizable evidence. Another important limitation is that, given the retrospective design, the exact quantity of insulin dose reduction could not be objectively determined for all patients. While patients reported whether they reduced their dose relative to baseline, the absolute number of units reduced could not be reliably obtained. Consequently, only the frequency rather than the magnitude of insulin dose reduction was reported—a limitation that would have been avoided in a prospective design. Furthermore, while the goal in T2DM management is to achieve the maximum semaglutide dose of 1.0 mg, only 29% of patients completing 30-week follow-up reached this dose. Escalation to 1.0 mg in a greater proportion of patients might have further influenced study outcomes. An additional important limitation of the present study is the absence of comprehensive data regarding diabetes duration, duration of insulin therapy, and the specific insulin regimens used by the patients. These variables may substantially affect the response to semaglutide treatment. Likewise, information regarding concomitant use of SGLT2 inhibitors and other antidiabetic medications that may influence weight reduction and HbA1c outcomes was limited. Inclusion of these data could have enhanced the scientific robustness and clinical relevance of the study.

## 5. Conclusions

In conclusion, this study provides real-world evidence from a specific geographic region of Türkiye that is consistent with prior studies and real-world data in patients with T2DM initiating semaglutide therapy. Semaglutide dose escalation yielded superior glycemic control and, critically, a significant reduction in insulin dose requirements without a meaningful increase in adverse events. This finding is of direct clinical relevance: by enabling insulin dose reduction, semaglutide dose escalation can reduce the risk of insulin-associated adverse events—particularly hypoglycemia and weight gain—while patients achieve their HbA1c targets. These results support escalating semaglutide to the maximum tolerated appropriate dose in patients with T2DM. Further large-scale multicenter studies involving more diverse patient populations in our country are necessary to better clarify and validate the clinical implications of our findings.

## Figures and Tables

**Table 1:** Baseline clinical, demographic and laboratory variables of patients according to semaglutide dose.

Variable	All Patients *n* = 500	0.25 mg (*n* = 97)	0.50 mg (*n* = 255)	1.00 mg (*n* = 148)	*p* Value
Age (years)	56.1 ± 10.8	55.9 ± 9.9	56.3 ± 10.7	55.8 ± 11.3	0.871
Men/women	255/245	51/46	131/125	73/75	0.729
Smoking, *n* (%)	106 (21.2%)	24 (24.7%)	45 (17.6%)	37 (25%)	0.342
Hypertension, *n* (%)	219 (43.8%)	46 (47.4%)	101 (39.6%)	72 (48.6%)	0.423
CAD, *n* (%)	61 (12.2%)	17 (17.5%)	29 (11.4%)	15 (10.1%)	0.074
CKD, *n* (%)	49 (9.8%)	13 (13.4%)	20 (7.8%)	16 (10.8%)	0.184
Dyslipidemia, *n* (%)	233 (46.6%)	42 (43.3%)	126 (49.4%)	65 (43.9%)	0.468
Weight (kg)	95.3 ± 15.2	93.9 ± 15.9	96.8 ± 15.3	93.6 ± 14.3	0.081
Waist circumference (cm)	96.2 ± 8.9	96.7 ± 8.9	96.4 ± 9.1	95.4 ± 8.5	0.481
BMI (kg/m^2^)	33.7 ± 3.9	33.6 ± 4.0	34.1 ± 3.9	33.3 ± 3.7	0.217
Glucose (mg/dL)	147 ± 19	144 ± 18 ^β^	146 ± 20	151 ± 19	**0.049**
HbA1c (%)	8.24 ± 0.94	8.11 ± 0.92	8.22 ± 0.91	8.36 ± 0.99	0.104
HbA1c < 7%, *n* (%)	38 (7.6%)	11 (11.3%)	19 (7.4%)	8 (5.4%)	0.138
Total cholesterol (mg/dL)	195 ± 28	193 ± 31	195 ± 27	197 ± 28	0.680
LDL cholesterol (mg/dL)	122 ± 24	122 ± 25	121 ± 23	124 ± 25	0.446
HDL cholesterol (mg/dL)	43.1 ± 7.7	43.5 ± 7.6	42.9 ± 7.8	42.9 ± 7.4	0.824
Triglyceride (mg/dL)	192 ± 58	200 ± 54	191 ± 62	190 ± 54	0.370
NT-proBNP (pg/mL)	253 ± 279	231 ± 170	253 ± 240	267 ± 380	0.599
uACR (mg/day)	54.6 ± 55.6	54.9 ± 50.8	55.1 ± 55.9	53.5 ± 58.3	0.959
Insulin treatment, *n* (%)	202 (40.4%)	34 (35.1%)	108 (42.4%)	60 (40.5%)	0.232
Treatment discontinued, *n* (%)	117 (23.4%)	21 (21.6%)	57 (22.4%)	39 (26.4%)	0.650
Due to GI side effect, *n* (%)	42 (8.4%)	4 (4.1%)	22 (8.6%)	16 (10.8%)	0.091
Non-compliance, *n* (%)	9 (1.8%)	0 (0%)	3 (1.2%)	6 (4.1%)	0.120
Other (cost, access), *n* (%)	66 (13.2%)	17 (17.5%)	32 (12.5%)	17 (11.5%)	0.201

Values are expressed as mean ± SD or n (%). Statistically significant *p* values are shown in bold. BMI: Body mass index; CAD: Coronary artery disease; CKD: Chronic kidney disease; GI: Gastrointestinal; HDL: High-density lipoprotein; LDL: Low-density lipoprotein; NT-proBNP: N-terminal pro-brain natriuretic peptide; uACR: Urinary albumin/creatinine ratio. β = significant difference between semaglutide 0.25 mg and 1.00 mg groups (*p* < 0.05).

**Table 2:** Follow-up clinical, medical treatment and laboratory variables according to semaglutide dose in patients continuing semaglutide treatment *.

Variable	Continued Patients *n* = 383	0.25 mg (*n* = 76)	0.50 mg (*n* = 198)	1.00 mg (*n* = 109)	*p* Value
Weight (kg)	88.8 ± 14.9	90.4 ± 16.0 ^β^	89.7 ± 14.9	85.8 ± 13.9	**0.045**
Absolute weight change (kg)	−6.65 ± 3.22	−4.21 ± 2.68 ^α,β^	−6.52 ± 2.84 ^¥^	−8.59 ± 2.98	**<0.001**
Weight loss ≥ 5%, *n* (%)	280 (73.1%)	33 (43.4%) ^α,β^	145 (73.2%) ^¥^	102 (93.6%)	**<0.001**
Weight loss ≥ 10%, *n* (%)	70 (18.3%)	2 (2.6%) ^α,β^	25 (12.6%) ^¥^	43 (39.4%)	**<0.001**
Waist circumference (cm)	92.9 ± 9.64	94.1 ± 9.7	93.1 ± 9.7	91.7 ± 9.4	0.251
BMI (kg/m^2^)	31.3 ± 3.86	32.1 ± 4.1 β	31.6 ± 3.9	30.4 ± 3.5	**0.010**
Δ—BMI change (kg/m^2^)	−2.35 ± 1.11	−1.49 ± 0.94 ^α,β^	−2.30 ± 0.98 ^¥^	−3.04 ± 1.01	**<0.001**
Glucose (mg/dL)	135 ± 23	134 ± 21	136 ± 24	134 ± 22	0.760
HbA1c (%)	7.21 ± 0.84	7.39 ± 0.83 ^β^	7.21 ± 0.85	7.10 ± 0.84	**0.042**
Absolute HbA1c change (%)	−1.03 ± 0.35	−0.72 ± 0.28 ^α,β^	−1.02 ± 0.26 ^¥^	−1.27 ± 0.34	**<0.001**
HbA1c < 7%, *n* (%)	169 (44.1%)	28 (36.8%) ^β^	87 (43.9%)	54 (49.5%)	**0.035**
Total cholesterol (mg/dL)	189 ± 32	199 ± 35	190 ± 31	182 ± 32	0.135
LDL cholesterol (mg/dL)	101 ± 23	109 ± 22 ^β^	103 ± 23	95.1 ± 20	**0.010**
Δ—LDL-C change (mg/dL)	−20.7 ± 14	−14.6 ± 8.7 ^α,β^	−18.9 ± 15 ^¥^	−28.1 ± 12	**<0.001**
HDL cholesterol (mg/dL)	43.9 ± 8.2	44.2 ± 7.5	43.6 ± 8.6	44.5 ± 8.1	0.625
Triglyceride (mg/dL)	182 ± 70	191 ± 65	184 ± 73	171 ± 66	0.154
NT-proBNP (pg/mL)	229 ± 287	204 ± 156	224 ± 218	257 ± 431	0.436
uACR (mg/day)	49.5 ± 52	52.1 ± 52	48.4 ± 52	49.6 ± 53	0.878
Insulin treatment, *n* (%)	144 (37.6%)	25 (32.9%)	78 (39.4%)	41 (37.6%)	0.344
Insulin dose reduction, *n* (%)	60 (41.7%)	10 (40%) ^α^	32 (41%)	21 (51%)	**0.010**
Insulin injection reduction, *n* (%)	60 (41.7%)	8 (32%) ^α^	30 (39%)	19 (46%)	**0.045**
OAD drug reduction, *n* (%)	111 (29%)	20 (26.3%)	59 (29.8%)	32 (29.4%)	0.399
Hypoglycemic episodes, *n* (%)	23 (6%)	5 (6.5%)	11 (5.5%)	6 (5.5%)	0.876
GI adverse effect, *n* (%)	141 (36.8%)	20 (26.3%) ^α^	67 (33.8%)	48 (44.1%)	0.054
Nausea, *n* (%)	98 (25.6%)	18 (23.7%)	44 (22.2%)	36 (33%)	**0.105**
Vomiting, *n* (%)	28 (7.3%)	5 (6.6%)	13 (6.6%)	10 (9.2%)	0.462
Diarrhea, *n* (%)	42 (11%)	7 (9.2%)	22 (11.1%)	13 (11.9%)	0.573
Constipation, *n* (%)	34 (8.9%)	3 (3.9%)	20 (10%)	11 (10%)	0.187

Values are expressed as mean ± SD or n (%). Statistically significant *p* values are shown in bold. BMI: Body mass index; GI: Gastrointestinal; HDL: High-density lipoprotein; LDL: Low-density lipoprotein; NT-proBNP: N-terminal pro-brain natriuretic peptide; OAD: Oral anti-diabetic drug; uACR: Urinary albumin/creatinine ratio. α = significant difference between semaglutide 0.25 mg and 0.50 mg groups (*p* < 0.05). β = significant difference between semaglutide 0.25 mg and 1.00 mg groups (*p* < 0.05). ¥ = significant difference between semaglutide 0.50 mg and 1.00 mg groups (*p* < 0.05). * Percentage calculated relative to insulin-treated patients only.

**Table 3:** Comparison between baseline and follow-up clinical and laboratory variables in patients continuing semaglutide treatment.

Variable	Baseline Variables *n* = 383	Follow-Up Variables *n* = 383	t and Z Value	*p*
Weight (kg)	95.5 ± 15.6	88.8 ± 14.9	**40.424**	**<0.001 ^a^**
Waist circumference (cm)	96.2 ± 8.9	92.8 ± 9.6	**18.433**	**<0.001 ^a^**
BMI (kg/m^2^)	33.7 ± 3.9	31.3 ± 3.9	**41.383**	**<0.001 ^a^**
Glucose (mg/dL)	147 ± 19	145 ± 22	**4.119**	**<0.001 ^a^**
HbA1c (%)	8.24 ± 0.95	7.21 ± 0.84	**58.644**	**<0.001 ^a^**
Total cholesterol (mg/dL)	195 ± 28	189 ± 32	**5.983**	**<0.001 ^a^**
LDL cholesterol (mg/dL)	121 ± 24	101 ± 23	**28.983**	**<0.001 ^a^**
HDL cholesterol (mg/dL)	42.9 ± 7.5	43.9 ± 8.2	**−5.611**	**<0.001 ^a^**
Triglyceride (mg/dL)	195 ± 59	182 ± 70	**6.800**	**<0.001 ^b^**
NT-proBNP (pg/mL)	249 ± 292	229 ± 287	**10.632**	**<0.001 ^b^**
uACR (mg/day)	54.9 ± 57.8	49.5 ± 52.1	**11.413**	**<0.001 ^b^**

Values are expressed as mean ± SD. Statistically significant *p* values are shown in bold. HDL: High-density lipoprotein; LDL: Low-density lipoprotein; NT-proBNP: N-terminal pro-brain natriuretic peptide; uACR: Urinary albumin/creatinine ratio. a: Paired *t*-test; b: Wilcoxon signed-rank test.

## Data Availability

The original contributions presented in this study are included in the article. Further inquiries can be directed to the corresponding author.

## Abbreviations

The following abbreviations are used in this manuscript:

ADA	American Diabetes Association
AEs	Adverse Events
BMI	Body Mass Index
CAD	Coronary Artery Disease
CKD	Chronic Kidney Disease
DPP-4	Dipeptidyl Peptidase-4
GI	Gastrointestinal
GLP-1RA	Glucagon-Like Peptide-1 Receptor Agonist
HbA1c	Glycated Hemoglobin
HDL	High-Density Lipoprotein
HT	Hypertension
LDL	Low-Density Lipoprotein
NT-proBNP	N-Terminal Pro-Brain Natriuretic Peptide
OAD	Oral Antidiabetic
SGLT-2	Sodium–Glucose Cotransporter-2
T2DM	Type 2 Diabetes Mellitus
TG	Triglyceride
uACR	Urinary Albumin/Creatinine Ratio
